# Higher cardiometabolic risk in idiopathic versus autoimmune type 1 diabetes: a retrospective analysis

**DOI:** 10.1186/s13098-018-0341-6

**Published:** 2018-05-10

**Authors:** Valentina Guarnotta, Enrica Vigneri, Giuseppe Pillitteri, Alessandro Ciresi, Giuseppe Pizzolanti, Carla Giordano

**Affiliations:** Biomedical Department of Internal and Specialist Medicine (DIBIMIS), Section of Diabetes, Endocrinology and Metabolism, University of Palermo, Italy, Piazza Delle Cliniche 2, 90127 Palermo, Italy

**Keywords:** Cardiovascular risk factors, Type 1 diabetes, c-Peptides, Insulin resistance, Insulin secretion

## Abstract

**Background:**

Idiopathic type 1 diabetes mellitus (IDM) is characterized by an onset with insulinopenia and ketoacidosis with negative β-cell autoimmunity markers and lack of association with HLA. The aim of the study is to compare the clinical and metabolic parameters, the macro and microvascular complications, the adipose tissue dysfunction and the insulin secretion and sensitivity indexes in patients with IDM and autoimmune type 1 diabetes mellitus (ADM) at clinical onset.

**Methods:**

Thirty patients with IDM and 30 with ADM, matched for age and gender, were retrospectively analyzed. BMI, waist circumference, lipids, glycemia, HbA1c, insulin requirement, glutamic oxaloacetic and glutamic pyruvic transaminases (GOT and GPT), glucagon stimulated c-peptide (GSC-pep) test levels, M value during hyperinsulinemic euglycemic clamp and Visceral Adiposity Index (VAI) were obtained from our database.

**Results:**

Patients with IDM showed a significantly higher BMI (p 0.012), WC (p 0.07), VAI (p 0.004), LDL-cholesterol (p 0.027), GOT (p 0.005), GPT (p 0.001), M value (p 0.006) and GSC-pep peak (p 0.036), with concomitant lower HDL-cholesterol (p < 0.001), than patients with ADM. In addition, patients with IDM showed a more marked familial history for diabetes (p 0.005) and a higher percentage of hepatic steatosis (p 0.001), visceral obesity (p 0.032) and hypercholesterolemia (p 0.007) compared to patients with ADM.

**Conclusions:**

Patients with IDM show many metabolic complications at onset, such as visceral obesity, hepatic steatosis and hypercholesterolemia and a higher cardiometabolic risk, than patients with ADM, similarly to patients with type 2 diabetes at onset.

## Background

Idiopathic type 1 diabetes mellitus (IDM) was first identified in 1984, when Ahren and Corrigan reported the existence of a subgroup of diabetic patients observed in Tanzania, where the need for insulin replacement therapy fluctuated with time, with waxes and wanes, and transient ketoacidosis developed [1]. In 1997 the American Diabetes Association, proposed two subcategories for type 1 diabetes mellitus (T1DM): classical autoimmune type 1 diabetes (ADM) and the IDM [2].

IDM is characterized by permanent insulinopenia without evidence of beta-cell autoimmunity [3]. Patients with IDM are prone to develop ketosis/ketoacidosis and exhibit various degrees of insulin deficiency between ketoacidosis episodes [4, 5]. IDM has been mostly described in African–American and Asian patients, even though it has also been described in native and Hispanic Americans and in European Mediterranean individuals [6, 7]. Although patients with IDM have generally an onset similar to that of patients with ADM, some differences are frequently found. Patients with IDM have a characteristic course of disease, with an initial requirement of insulin therapy, generally for a period going from 6 to 18 months, and subsequent good control of metabolic disease with oral agents [6, 8–10]. They show a different phenotype when compared to patients with ADM, generally characterized by visceral obesity at the time of diagnosis, and have a major family history of type 2 diabetes mellitus [11]. On the other side, patients with ADM are generally lean and young, with autoimmune markers associated with diabetes, and most of them have susceptibility for specific HLA haplotypes [12]. They show rapid β-cell destruction mediated by T-cells, and need persistent exogenous insulin to preserve their lives.

Due to the presence of some metabolic features of type 2 diabetes, IDM has also been referred to in the literature as atypical diabetes, type 1.5 diabetes, Flatbush diabetes and ketosis-prone diabetes [9, 13–15]. Information about IDM pathogenesis is scarce and not exhaustive. It may be related to insulin resistance and transient β-cell dysfunction, due to glucolipotoxicity and lipotoxicity mechanisms [9, 16]. HLA (human leukocyte aplotype)-related genes are not believed to be involved in its pathogenesis, even though mutations in different genes from HLA have been reported, suggesting that IDM may have a specific genetic background [17, 18]. The aim of the current study is to compare clinical and metabolic parameters, β-cell pancreatic function and adipose tissue dysfunction in patients with IDM and ADM.

## Methods

### Subjects

This pilot study was approved by the Institutional Review Board at the Faculty of Medicine of the University of Palermo. At the time of observation all patients regularly signed an informed consent for the scientific use of their data. Thirty consecutive patients with IDM presenting in our Clinic with unprovoked ketoacidosis (not associated with precipitating events, such as infections, trauma, acute pancreatitis, etc.), from January 2005 to December 2015, were retrospectively studied. To be eligible, patients needed to fulfill some clinical characteristics: (1) typical symptoms of diabetes before admission (polyuria, polydipsia, polyphagia and weight loss); (2) diabetic ketoacidosis (anion gap > 12 mEq/l plus Ph < 7.35 and/or HCO_3_ < 17 mEq/l) and urine ketones > 80 mg/dl; (3) absence of immune markers, such as islet cell antibodies (ICA), insulin antibodies (IA), glutamic acid decarboxylase antibodies (GADA), islet antigen-2 antibodies (IA-2A) at the diagnosis and over one-year from the onset of diabetes. Zinc transporter 8 antibody (ZnT8A) was assayed only in 24 out of 30 patients with IDM, those with more recent diagnosis.

Twenty out of 30 patients were not Caucasian: 11 out of 20 were of African–American origin, while 9 were of Indian origin.

Among the Sicilian patients consecutively hospitalized in our Division from January 2005 to December 2015 for ADM, we selected, as a control group, 30 patients, matched for gender (10 F, 20 M) and age at diagnosis (mean age 30.4 ± 6.5 years), who were treatment-naïve, with specific beta-cell autoimmunity and with ketosis or ketoacidosis at onset and/or polyuria for more than 6 months before onset.

All patients included in this study were regularly treated with conventional basal-bolus therapy.

The following relevant data were obtained from our databases after correction of acute hyperglycemia and metabolic profile, after a mean of 30–40 days after the onset: family history of type 2 diabetes, arterial hypertension, diabetic retinopathy, nephropathy and/or neuropathy, coronary heart disease, peripheral and cerebral vascular disease, hepatic steatosis (evaluated by liver echography), visceral obesity and hypercholesterolemia, BMI, waist circumference (WC), systolic (SBP) and diastolic (DBP) blood pressure, lipids, fasting glycemia, HbA1c, insulin requirement, glutamic oxaloacetic and glutamic pyruvic transaminases (GOT and GPT) and pancreatic β-cell function.

Pancreatic β-cell function was assessed in all patients by means of a glucagon stimulated c-peptide (GSC-pep) test at diagnosis (after correction of ketosis or ketoacidosis and stabilization of blood glucose levels). Basal and GSC-pep were assessed in the morning after an overnight fasting period of 8–10 h. Patients took no short-acting insulin for at least 6 h and bedtime long-acting insulin at least 12 h before the test. The test was performed only if fasting glucose was 72–200 mg/dl (4–11.1 mmol/l). 1 mg of glucagon was intravenously injected within 10 s [19, 20]. Samples were drawn at 1, 3, 6, 10 and 15 min after the end of glucagon administration.

Data about euglycaemic hyperinsulinaemic clamp, used to evaluate insulin sensitivity, were also extracted. The clamp procedure was performed as follows, after patients reached a glycaemic stabilization. One catheter was placed in a vein on the forearm for administration of insulin and glucose and the second catheter was placed in a vein of the contralateral forearm for blood samples. The clamp was performed under standard conditions, i.e. the plasma insulin concentration was acutely raised with insulin priming (0–3 min: 113.6 mU/m^2^, 3–6 min: 80.2 mU/m^2^, 7–10 min: 50.4 mU/m^2^ of body surface area) for the first 10 min of the test and maintained by continuous infusion of insulin infusion (40 mU/m^2^ for the remaining 110 min). The rate of peripheral glucose utilization (M value) was calculated by dividing the glucose amount infused during the last 40 min by body weight measured in kilograms (milligrams per kilogram per minute). The plasma glucose concentration was held constant at normoglycaemic levels (values between 4.4 and 7.22 mmol/l) by variable glucose infusion, and under the steady state conditions of euglycemia the glucose infusion rate equaled glucose uptake by all the tissues in the body and was therefore considered a measure of tissue sensitivity to exogenous insulin [21].

As an indicator of adipose tissue function, the Visceral Adiposity Index (VAI) was calculated according to gender, where TG is triglycerides levels expressed in mmol/l and HDL is HDL-cholesterol levels expressed in mmol/l:

Males, VAI = [WC/39.68 + (1.88 × BMI)] × (TG/1.03) × (1.31/HDL);

Females, VAI = [WC/36.58 + (1.89 × BMI)] × (TG/0.81) × (1.52/HDL) [22].

### Assays

Insulin, glycemia, c-peptide, aspartate and alanin aminotransferase and lipids were measured by standard methods (Modular P800, Roche, Milan). LDL-C levels were measured using the Friedewald formula [total cholesterol − (HDL + (TG/5)]. HbA1c levels were determined by HPLC with an ion-exchange resin (Bio-Rad Laboratories, Milan, Italy).

The conversion factors for the International System were the following: glucose (mg/dl vs. mmol/l: 0.0555), total and HDL cholesterol mg/dl vs. mmol/l: 0.0259, triglycerides mg/dl vs. mmol/l: 0.0113, c-peptide ng/dl vs. nmol/l: 0.33.

### Statistical methods

SPSS version 17 and MedCalc version 11.3 were used for data analysis. Patients’ characteristics were presented as mean ± SD for continuous variables; rates and proportions were calculated for categorical data. Normality of distribution for quantitative data was assessed by the Shapiro–Wilk test. The differences between patients with IDM and ADM and patients with IDM in groups A and B were detected by the unpaired Student’s t test for continuous variables (after testing for equality of variance: Levene test) and by the χ^2^ test and Fisher’s exact test (when appropriate) for categorical variables. Simple univariate correlations among continuous variables with normal distribution were determined by Pearson’s test. To evaluate the independent variables influencing the M value, a linear regression model was performed. A p value of ≤ 0.05 was considered statistically significant.

## Results

Patients with IDM were 8 females and 22 males, mean age at diagnosis was 33.1 ± 7.6 years. Among them 20 out of 30 patients were not Caucasian: 11 out of 20 were of African–American origin, while 9 were of Indian origin. On the other side, patients with ADM were 10 females and 20 males, with mean age at diagnosis of 30.4 ± 6.5 years.

No clinical and metabolic differences were found among the different ethnic groups in patients with IDM (data not shown). Over 1-year from the onset of diabetes no patients with IDM had positive autoimmune markers. In addition, after 1 year of treatment 12 out of 30 continued insulin with a significant decrease of the insulin requirement, than baseline (group A), while the remaining 18 out of 30 patients with IDM, discontinued the insulin therapy (group B).

### Clinical and metabolic characteristics and β-cell pancreatic function in patients with IDM and ADM

Patients with IDM showed significantly higher BMI (p = 0.012), WC (p = 0.07), VAI (p = 0.004), LDL-cholesterol (p = 0.027), GOT (p = 0.005), GPT (p = 0.001), M value (p = 0.006) with concomitant lower HDL-cholesterol (p < 0.001), than patients with ADM (comparison between means) (Table 1).

**Table 1:** Clinical and metabolic parameters in patients with idiopathic and autoimmune type 1 diabetes at onset, after metabolic stabilization

	Idiopathic type 1 diabetes (N = 30)	Autoimmune type 1 diabetes (N = 30)	p*
	Mean ± SD	Mean ± SD	
Clinical parameters			
BMI (kg/m^2^)	25.5 ± 3.06	23.1 ± 3.46	0.012
WC (cm)	97.8 ± 10.1	89.4 ± 11.1	0.007
SBP (mmHg)	112.6 ± 18.7	107.3 ± 20.1	0.104
DBP (mmHg)	68.8 ± 9.2	68.5 ± 8.1	0.876
VAI	1.76 ± 0.43	1.01 ± 0.49	0.004
Metabolic parameters			
HDL-cholesterol (mmol/l)	0.99 ± 0.11	1.37 ± 0.26	< 0.001
Triglycerides (mmol/l)	0.89 ± 0.26	0.94 ± 0.31	0.668
LDL-cholesterol (mmol/l)	3.47 ± 0.75	2.95 ± 0.68	0.027
Total-cholesterol (mmol/l)	4.88 ± 0.78	4.75 ± 0.73	0.509
GOT (U/l)	22.1 ± 8.81	16.4 ± 3.72	0.005
GPT (U/l)	22.8 ± 9.58	15.3 ± 5.57	0.001
Glycaemia (mmol/l)	7.16 ± 0.98	7.4 ± 1.02	0.190
HbA1c (%)	8.34 ± 1.17	8.01 ± 0.92	0.233
Insulin requirement (U/kg)	0.51 ± 0.21	0.67 ± 0.32	0.678
Insulin Sensitivity Index			
M value	3.13 ± 1.06	3.95 ± 0.98	0.006

* *p* comparison between means in patients with idiopathic and autoimmune type 1 diabetes

On the other side, no difference in SBP (p = 0.104) and DBP (p = 0.876), TG (p = 0.668), glycemia (p = 0.295), HbA1c (p = 0.812) and insulin-requirement (p = 0.678) was found between patients with IDM and ADM (comparison between means) (Table 1).

**Fig. 1:**
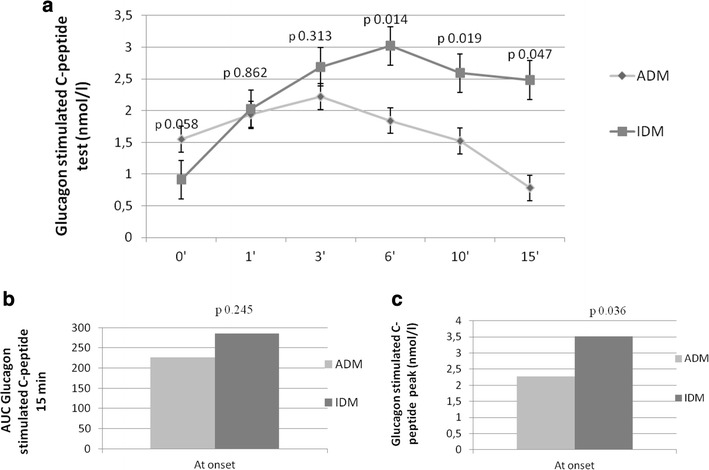
**a** Beta cell function assessed through glucagon stimulated C-peptide (GSC-pep) test at times 0–1–3–6–10 and 15 min in patients with idiopathic (IDM) and autoimmune (ADM) type 1 diabetes at onset, after metabolic stabilization. **b** Area under curve (AUC) of GSC-pep 15 min in patients with IDM and ADM at onset, after metabolic stabilization. C. GSC-pep peak in patients with IDM and ADM at onset, after metabolic stabilization

With regard to β-cell pancreatic function, no difference was found in basal c-peptide and the area under curve (AUC) during GSC-pep (AUC_GSC-pep 15 min_) (Fig. 1a, b) (comparison between means). However, patients with IDM had a significantly higher GSC-pep peak compared to patients with ADM (p = 0.036) (comparison between means) (Fig. 1a, c).

### Macro and microvascular and metabolic complications in patients with IDM and ADM

Patients with IDM showed higher prevalence of hepatic steatosis (p = 0.001), visceral obesity (p = 0.032) and hypercholesterolemia (p = 0.007) compared to patients with ADM (comparison between categorical variables) (Table 2).

**Table 2:** Macro and microvascular and metabolic complications of patients with idiopathic and autoimmune type 1 diabetes mellitus at onset, after metabolic stabilization

	Idiopathic type 1 diabetes (N = 30)	Autoimmune type 1 diabetes (N = 30)	p*
	Subjects (%)	Subjects (%)	
Family history of type 2 diabetes	12 (40%)	9 (30%)	0.430
Macrovascular complications			
Arterial hypertension	2 (6%)	4 (13%)	0.389
Coronary heart disease	0	0	/
Peripheral vascular disease	0	0	/
Cerebral vascular disease	0	0	/
Microvascular complications			
Diabetic retinopathy	2 (6%)	0	0.150
Diabetic nephropathy	2 (6%)	4 (13%)	0.389
Diabetic neuropathy	2 (6%)	0	0.150
Metabolic complications			
Hepatic steatosis	9 (30%)	0	0.001
Visceral obesity	15 (50%)	7 (23%)	0.032
Hypercholesterolemia	16 (53%)	6 (20%)	0.007

* *p* comparison between categorical variables in patients with idiopathic and autoimmune type 1 diabetes

No significant difference in family history of diabetes, arterial hypertension, diabetic retinopathy, nephropathy and neuropathy, coronary heart disease, peripheral and cerebral vascular disease was observed comparing patients with IDM and ADM (Table 2).

### Correlations of M-value with anthropometric and metabolic parameters

M-value showed a positive correlation with gender (p = 0.007; r 0.319), AUC_GSC-pep 15 min_ (p < 0.001; r 0.432), GSC-pep peak (p < 0.001; r 0.472) and an inverse correlation with LDL-cholesterol (p = 0.039; r − 0.246), and presence of autoantibodies (p < 0.001; r − 0.442). At the multivariate analysis, male gender (p = 0.013), AUC_GSC-pep 15 min_ (p = 0.001) and presence of autoantibodies (p < 0.001) were independently associated with M value (Table 3).

**Table 3:** Baseline variables independently associated with the M value at multivariate analysis (multiple linear regression)

Independent variable	Dependent variable			
	*β*	*t*	*p*	95% CI
Male gender	0.319	0.2560	0.013	0.028 to 0.231
AUC_GSC-pep 15 min_	0.432	3.643	0.001	29,548 to 101,625
Presence of autoantibodies	− 0.442	0.3748	< 0.001	− 0.553 to 0.205

### Clinical and metabolic characteristics and β-cell pancreatic function in patients with IDM (groups A and B)

Patients of group B showed significant higher values of VAI (p < 0.001) and TG (p = 0.017), than group A (comparison between means) (Table 4).

**Table 4:** Patients with idiopathic diabetes divided in groups A (patients who continued insulin after 12 months from the disease onset) and B (patients who suspended insulin after 12 months from the disease onset)

	Group A (N = 12)	Group B (N = 18)	p*
	Mean ± SD	Mean ± SD	
Clinical parameters			
Age of onset	31.3 ± 7.31	26.8 ± 10.7	0.220
BMI (kg/m^2^)	25.7 ± 3.01	24.9 ± 3.31	0.529
WC (cm)	97.4 ± 11.3	97.3 ± 9.8	0.994
SBP (mmHg)	112.9 ± 14.9	112.6 ± 8.83	0.953
DBP (mmHg)	69.6 ± 9.14	67 ± 9.84	0.461
VAI	1.09 ± 0.22	1.71 ± 0.34	< 0.001
Metabolic parameters			
HDL-cholesterol (mmol/l)	1.01 ± 0.31	0.98 ± 0.08	0.540
Triglycerides (mmol/l)	0.77 ± 0.11	0.98 ± 0.27	0.017
LDL-cholesterol (mmol/l)	3.41 ± 0.67	3.52 ± 0.81	0.689
Total-cholesterol (mmol/l)	4.78 ± 0.67	4.96 ± 0.85	0.529
GOT (U/l)	25.5 ± 10.4	19.8 ± 6.60	0.082
GPT (U/l)	25.9 ± 9.63	19.3 ± 9.35	0.075
Glycaemia (mmol/l)	7.47 ± 0.90	6.95 ± 1.01	0.156
HbA1c (%)	8.01 ± 1.26	8.56 ± 1.09	0.216
Insulin requirement (U/kg)	0.57 ± 0.10	0.46 ± 0.22	0.129
Insulin Sensitivity Index			
M value	3.4 ± 1.03	2.63 ± 1.12	0.071

* *p* comparison between means in patients with idiopathic type 1 diabetes, groups A and B

**Fig. 2:**
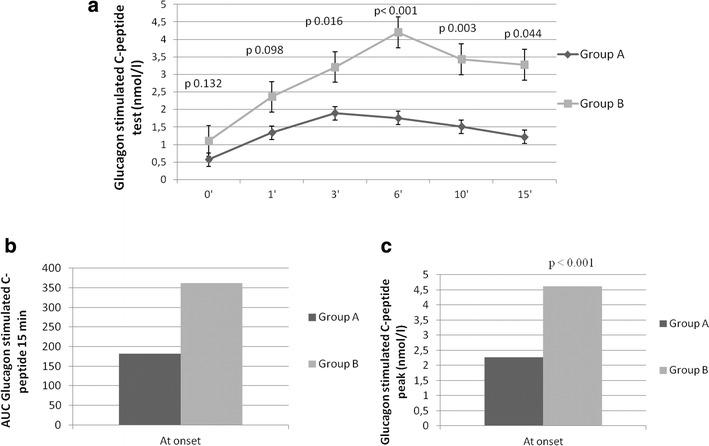
**a** Beta cell function assessed through glucagon stimulated C-peptide (GSC-pep) test at times 0–1–3–6–10 and 15 min in groups A and B. **b** Area under curve (AUC) of GSC-pep 15 min in groups A and B. **c** GSC-pep peak in patients with IDM and ADM in groups A and B

With regard to β-cell pancreatic function, significant higher c-peptide during GSC-pep at 6 (p = 0.016), 10 (p < 0.001) and 15 min (p = 0.003), AUC_GSC-pep 15 min_ (p = 0.005) and GSC-pep peak (p = 0.001) were found in group B than A (comparison between means) (Fig. 2).

## Discussion and conclusions

The current study shows that, despite an initial clinical presentation similar to that of classical ADM, patients with IDM differ for some specific characteristics such as the presence of visceral obesity, lower insulin sensitivity, hepatic steatosis and atherogenic lipid profile, and appear to show higher cardiometabolic risk.

We found a 2.5:1 man-to-woman ratio in patients with IDM, in line with previous studies, which reported a 2:1 man-to-woman ratio, notably in patients aged 15–35 years [7], supporting the high male predominance. The mean age at diabetes onset, in the current study, was lower than that reported in a recent study [23], but consistent with a previous study [6]. In line with previous studies [14, 24–29], IDM was associated with higher visceral obesity than ADM, but with a clinical phenotype similar to that of patients with type 2 diabetes mellitus [23, 25, 30, 31, 43].

Patients with IDM showed hepatic steatosis and the typical atherogenic lipid profile, characterized by high LDL-cholesterol and low HDL-cholesterol, lower insulin sensitivity (M value) and higher levels of VAI than patients with ADM, showing that IDM is associated with stronger insulin resistance and higher cardiometabolic risk than ADM [32].

The term cardiometabolic risk was coined by the American Diabetes Association [33] and the American Heart Association [34] to describe the overall risk of developing type 2 diabetes and cardiovascular diseases. Visceral obesity, insulin resistance and “dysfunctional” visceral fat are strong components of a constellation of metabolic abnormalities, related to energy surplus (due to the combination of a sedentary lifestyle and excessive calorie consumption), characterizing “cardiometabolic risk” [35, 36]. To predict the visceral adipose tissue-associated cardiometabolic risk [37], the VAI has been demonstrated to be a useful tool for the assessment of visceral adipose dysfunction and cardiometabolic risk and has been demonstrated to strongly correlate with adipokine profile in patients with type 2 diabetes [38]. Among all the anthropometric indexes studied, the VAI is the one that showed the greatest number of correlations with the adipocytokines and the lipids, including non-esterified fatty acids (NEFA) [35]. The VAI was able to express both the altered endocrine function of adipose tissue and the state of relative leptin resistance and low-grade inflammation, which are all alterations present in a state of adipose tissue dysfunction. Particularly strong was the correlation between VAI and visfatin, adipocytokine prevalently secreted by visceral adipose tissue, which according to some authors may have adipogenic effects and is a good candidate to explain the accumulation of visceral adipose tissue associated with insulin resistance [39], resistin, a potential mediator of obesity related insulin resistance in rodents, but still today a subject of controversy in human obesity and human epidemiologic studies [40] and hs-CRP, systemic markers of inflammation, which have been found to independently predict future coronary heart disease in type 2 diabetes [41]. In addition, VAI has proven to be the only index that showed an inverse correlation with adiponectin levels [38]. This datum, also confirmed in other studies [42], was of major significance, given that adiponectin reduction is a key player in the development of cardiovascular complications in type 2 diabetes, being the only known protective adipocytokine with insulin-sensitizing, anti-inflammatory and anti-atherogenic properties [43].

The application of the VAI as a marker of cardiometabolic risk could have some limitations, mainly relating to the presence of variables in the model that may change over time in relation to lifestyle and/or pharmacological treatment. Therefore, the VAI is an indicator of altered adipose function associated with cardiometabolic risk, but without a predictive future role. Prospective large-scale studies aiming to consider the possible predictive value of the VAI regarding cardiovascular risk must necessarily take into account its variation over time and need to be confirmed in patients with type 1 diabetes.

As expected, patients with IDM showed more insulin resistance than patients with ADM, demonstrated by M values. In the current study, we found a positive correlation between male gender and M value and AUC_GSC-pep 15 min_ and M value. Thus, a decreased insulin sensitivity was influenced by the male gender and by high secretion of c-peptide. In addition, the presence of autoantibodies was negatively correlated with M value. However, despite the higher insulin resistant phenotype, patients with IDM did not show different insulin requirement than ADM, after the metabolic stabilization, maybe related to the higher c-peptide secretion.

Indeed, with regard to β-cell pancreatic function, patients with IDM showed no differences in basal c-peptide and AUC_GSC-pep 15 min_, compared to patients with ADM. However, a higher beta-cell response to glucagon was observed in patients with IDM than ADM. This interesting finding may suggest a functional insulin secretory defect rather than a reduced beta-cell mass at diabetes onset, even though the key factors involved in the impairment of β-cell secretory capacity in people with IDM warrant further investigation. Similar findings have been reported by other previous studies conducted on a European Mediterranean population [7]. Interestingly, other studies reported a severe insulin secretory deficiency only during the acute ketotic phase in patients with IDM, demonstrating that this deficiency was associated with basal hyperglucagonemia and reduced c-peptide response to glucagon [14, 25]. Also, the clinical remission phase correlated to a restoration in insulin secretion in response to the same stimuli, but was lower than in healthy controls [14, 25, 44]. In our study, we confirmed insulin secretory deficiency at disease onset, even though patients with IDM maintained a higher c-peptide peak in response to glucagon than patients with ADM, suggesting the hypothesis of a possible recovery of β-cell function in the subsequent remission phase. Interestingly, among patients with IDM, those who suspended insulin after 12 months from the disease onset, showed at the diagnosis higher values of c-peptide than patients who continued insulin, supporting further the importance of c-peptide secretion in the evolution of IDM.

There are some limitations of this study that should be mentioned, such as the sample size and the time of evaluation. Indeed, a bigger study population may be helpful to further stratify the study population based on specific phenotypes, and a longer evaluation may clarify some further metabolic aspects of the disease. A second limitation was the evaluation of patients with different ethnic origins. Also, glucagon levels and incretin tone were not evaluated, due to the retrospective analysis of the patients’ data. However, many previous studies have evaluated the glucagon secretion and in some of them no significant difference in glucagon secretion in response to arginine was found comparing patients with IDM and controls, suggesting that the islet α-cell response to insulin may be preserved [44].

However, the strength of the study is the evaluation of insulin sensitivity, by clamp, and secretion, by glucagon test, in the same cohort of patients and the application of the VAI as an indicator of cardiometabolic risk, never evaluated before in this context.

In conclusion, patients with IDM show many metabolic complications at onset, such as visceral obesity, hepatic steatosis and hypercholesterolemia, despite an initial phase of ketoacidosis that may be associated with an acute decreased β-cell secretion and sensitivity. They show higher adipose tissue dysfunction than patients with ADM, which exposes them to a stronger cardiometabolic risk even at disease onset and needs to be diagnosed early and adequately treated to reduce the risk of cardiovascular complications in the long term.

## Abbreviations

IDM: idiopathic diabetes mellitus
T1DM: type1 diabetes mellitus
ADM: autoimmune diabetes mellitus
ICA: islet cell antibodies
IA: insulin antibodies
GADA: glutamic acid decarboxylase antibodies
IA-2A: islet antigen-2 antibodies
ZnT8A: zinc transporter 8 antibody
WC: waist circumference
SBP: sistolic blood pressure
DBP: diastolic blood pressure
GSC-pep: glucagon stimulated c-peptide
VAI: Visceral Adiposity Index
TG: triglycerides
AUC: area under the curve
NEFA: not esterified fatty acids
